# Aspirin desensitization for urgent endovascular treatment of the aneurysm

**DOI:** 10.1186/2045-7022-4-S3-P25

**Published:** 2014-07-18

**Authors:** Rita Aguiar, Natália P Fernandes, Ana Mendes, Elisa Pedro, Manuel Pereira-Barbosa

**Affiliations:** 1 Hospital Santa Maria, CHLN, Immunoallergology Department, Portugal

## 

Hypersensitivity reactions to aspirin (acetylsalicylic acid- ASA) and other nonsteroidal anti-inflammatory drugs (NSAIDs) constitute only a subset of all adverse reactions to these drugs, but due to their severity they pose a significant risk to patients. The authors report a case of a 48 year old woman with asthma, hypertension, obstructive sleep apnea and a NSAIDs-exacerbated respiratory disease: hypersensitivity reactions induced by aspirin and metamizol manifesting primarily as bronchial obstruction and dyspnea. She was regulary medicated with budesonide 400mg 2x/day, ipratropium bromide dry powder inhaler once a day, montelukast 10mg/day, bilastine 20mg/day and terbutaline. The patient had a giant fusiform aneurysm with carotid-cavernous sinus partially thrombosed in a subacute phase. For endovascular treatment of aneurysm, the patient need a stent placement in the left internal carotid artery under antiplatelet therapy with ASA. The patient was referred to our Department and was submitted to a desensitization protocol in an emergence context due to the risk of aneurism rupture. At the time, her asthma was not controlled so it was necessary to carry out medication with bronchodilators and oral corticosteroids while doing the procedure. The protocol followed this sequence: on the first day 5, 10, 25, 50, 75, 100 and 250mg of ASA were administered at intervals of 30 minutes. On the second day the patient performed one single dose of 500mg, without reactions. After surgery, she remained on 100mg of ASA daily without reaction. The authors sought to adapt a safe and practical protocol to allow the administration of ASA to patients with a NSAIDs-exacerbated respiratory disease. In live-saving situations is very important to reflect, reevaluate the indications and contraindications for desensitization. Note that it is possible to make a successful desensitization even in a patient with uncontrolled asthma.

